# Daytime Dysfunction: Symptoms Associated with Nervous System Disorders Mediated by SIRT1

**DOI:** 10.3390/biomedicines12092070

**Published:** 2024-09-11

**Authors:** Tianke Huang, Xianxie Zhang, Ling Qi, Fang Li, Zuoxu Liu, Zhixing Wang, Yi Ru, Maoxing Li, Chengrong Xiao, Yuguang Wang, Zengchun Ma, Yue Gao

**Affiliations:** 1 School of Pharmacy, Guangdong Pharmaceutical University, Guangzhou 510006, China; huangtianke2022@163.com (T.H.); wangyg@bmi.ac.cn (Y.W.); mazchun@139.com (Z.M.); 2 Department of Pharmaceutical Sciences, Beijing Institute of Radiation Medicine, Beijing 100850, China; 3 State Key Laboratory of Kidney Diseases, Chinese PLA General Hospital, Beijing 100853, China

**Keywords:** daytime dysfunction, sleep–wake, memory and cognition, SIRT1

## Abstract

Daytime dysfunction, including symptoms like sleepiness, poor memory, and reduced responsiveness, is not well researched. It is crucial to develop animal models and study the biological mechanisms involved. We simulated sleep disorders through sleep deprivation, and stressful stimuli were used to establish daytime functional animal models. We used tests like the sodium pentobarbital sleep synergy test and the DSI telemetry system to measure sleep duration and structure. We also used tests like the Morris water maze, open field test, grip test, and baton twirling test to assess mental and physical fatigue. To assess the intrinsic biological mechanisms, we measured sleep–wake-related neurotransmitters and related receptor proteins, circadian rhythm-related proteins and cognition-related proteins in hypothalamus tissue, and oxidative stress, inflammatory factors, S100β, and HPA axis-related indexes in serum. Multi-factor sleep deprivation resulted in the disruption of sleep–wakefulness structure, memory–cognitive function degradation, decreased grip coordination, and other manifestations of decreased energetic and physical strength. The intrinsic biological mechanisms were related to the disturbed expression of sleep–wake, circadian rhythm, memory–cognition-related proteins, as well as the significant elevation of inflammatory factors, oxidative stress, the HPA axis, and other related indicators. Intrinsically related biological mechanisms and reduced sirt1 expression can lead to disruption of circadian rhythms; resulting in disruption of their sleep–wake-related neurotransmitter content and receptor expression. Meanwhile, the reduced expression of sirt1 also resulted in reduced expression of synapse-associated proteins. This study prepared an animal model of daytime dysfunction by means of multi-factor sleep deprivation. With sirt1 as a core target, the relevant biological mechanisms of neurological disorders were modulated.

## 1. Introduction

Daytime dysfunction, as defined in entry 7 of the Pittsburgh Sleep Quality Index (PQSI), is a cluster of symptoms arising from poor sleep quality or other conditions, critical for daily activities and operational efficiency, especially high-precision tasks. It includes symptoms like fatigue, poor concentration, memory issues, mood changes, daytime sleepiness, low energy, increased errors and accidents, excessive focus on sleep, and dissatisfaction with sleep quality, all of which can impact daily work and life. Studies have shown that more than one in five people have daytime sleepiness the next day due to sleep problems [1]. People with sleep disorders have a 1.68 times higher risk of cognitive decline and Alzheimer’s disease, and the risk is even higher in some specific conditions [2]. At the same time, severe insomnia was strongly associated with comorbidities of mental illness, with the prevalence of any mental illness at 37.4% and depression at 21.7%, while the prevalence was lower in people without sleep problems [3].

These symptoms, collectively known as daytime dysfunction, are a mix of neurological disorders that have received little holistic research attention [4,5,6].

Wakefulness is essential for all human and animal activities. Regular sleep–wake patterns are crucial for maintaining physiological functions and daily activities, especially those related to social interactions and work [7,8]. Modern fast-paced lifestyles and high stress levels lead to widespread sleep deprivation and mental stress. Epidemiological surveys indicate that 30–48% of the global population suffers from sleep problems, with this number increasing [5,8,9].

Consequently, daytime dysfunction due to sleep disorders is also rising. The PQSI is a crucial tool for patients to self-assess their sleep quality, where higher scores indicate more severe daytime dysfunction [5,10].

Sleep deprivation significantly impairs memory, alertness, cognition, and attention, while high work–life stress fosters negative emotions and behaviors, impacting overall health, in which the body is seen as a unified whole [11,12,13]. Daytime dysfunction symptoms like drowsiness and memory loss are termed “shenpi” (divine fatigue), while lack of physical strength is called “tilao” (physical exertion). Meanwhile, there is a bidirectional relationship between sleep and metabolism, recognizing that sleep disturbances can lead to metabolic dysregulation, and metabolic disorders can also affect sleep quality.

Creating animal models of daytime dysfunction should integrate these symptoms to reflect the complex nature of the condition and explore the underlying biological mechanisms [14].

## 2. Materials and Methods

### 2.1. Chemicals and Reagents

Caffeine was obtained from Mian Site (Chendu, China). Assay Kits for malondialdehyde (MDA), glutathione (GSH), superoxide dismutase (SOD), tumor necrosis factor-alpha (TNF-α), interleukin (IL)-6 (IL-6), interleukin (IL)-1β(IL-1β), corticotropin-releasing hormone (CRH), adrenocorticotropic hormone (ACTH), corticosterone (CORT), and S100β were purchased from Meimian (Yancheng, China). Radio immunoprecipitation assay (RIPA) lysis buffer and the BCA protein detection kit were purchased from Epizyme Biotech (Shanghai, China). Antibodies against CLOCK were purchased from Thermofisher (Boston, MA, USA); BAML1, SIRT1, Cry2, Per2, and 5-HT1A were purchased from Abcam (Cambridge, UK); orexinR2 and postsynaptic density protein-95 (PSD95) were purchased from proteintech (Wuhan, China); anti-rabbit horseradish peroxidase (HRP)-conjugated IgG was purchased from Cell Signal Technique (Boston, MA, USA). Alanine aminotransferase (ALTL), aspartate aminotransferase (ASTL), creatinine (CREJ2), creatine kinase-MB2 (CK-MB2), lactate dehydrogenase (LDHI2), total protein (TP2), triglyceride (TRIGL), and lecithin-cholesterolacyltransferase (LACT2) were obtained from Roche Diagnostics (Mannheim, Germany).

### 2.2. Animals Model

Animal selection: Although rodent polyphasic sleep patterns differ significantly from human monophasic sleep and their activity rhythms are opposite to those of humans, both display a circadian rhythm with light time and dark time. The model can cause a decrease in activity levels during the dark time, a reduction in wakefulness and wake duration, impaired memory and cognitive functions, reduced activity and motor balance, and depressive mood, which are consistent with human daytime dysfunction [15,16,17].

Male C57BL/6J mice weighing 20 ± 2 g and male SD rats weighing 220 ± 20 g were purchased from Beijing Weitong Lihua Experimental Animal Technology Co., Ltd. (Beijing, China). Mice and rats were housed in micro isolator cages under a 12 h light–dark cycle with un limited access to food and water under standard laboratory conditions. All animal experiments were performed with the approval of the intra mural Committee on Ethics Conduct of Animal Studies of the Academy of Military Medical Sciences, China, with ethical approval no. IACUC-DWZX2020–762.

Single-factor sleep deprivation experiment: 72 h of sleep deprivation (rotating rod sleep deprivation; instrument model: XR-XS108 (Xinruan Information Technology, Co., Ltd., Shanghai, China); parameters set at 42 s per rotation, one rotation clockwise and one counterclockwise).

Mice were randomly divided into three groups (*n* = 8 per group): negative control group (NC), single-factor sleep deprivation (S-SD), caffeine intervention in single-factor sleep deprivation (S-COF). Animals in all groups except the NC group were subjected to single-factor sleep deprivation.

Multi-factor sleep deprivation experiment: sleep deprivation accompanied by circadian reversal for 72 h (rotating rod sleep deprivation; instrument model: XR-XS108; parameters set at 42 s per rotation, one rotation clockwise and one counterclockwise). At the same time, electrical stimulation of 0.5 mA/2 s was performed in the first 24 h of sleep deprivation, and cold water stimulation was performed every 24 h, a total of 3 times.

Mice were randomly divided into three groups (*n* = 8 per group): negative control group (NC), multi-factor sleep deprivation (M-SD), caffeine intervention in multi-factor sleep deprivation (M-COF). Animals in all groups except the NC group were multi-factorially sleep-deprived. The animals were given caffeine by gavage between 9 and 10 am every day. The NC group and S-SD group/M-SD group were given the same volume ratio of pure water by intragastric administration. After 72 h of sleep deprivation, behavioral experiments and tissue collection were performed.

### 2.3. Behavioral Evaluation

#### 2.3.1. Pentobarbital Sodium Sleep Synergistic Experiment

Sleep is marked by loss of the righting reflex. If more than 30~60 s cannot be righted, that is, the righting reflex disappears, sleep is entered. The recovery of the righting reflex represents the animal awakening. 

The period from the disappearance of righting reflex to the recovery was the sleep time of the animals. The sleep time and wake time of each group were recorded. Sleep latency was documented from the time of pentobarbital injection to the time of sleep onset, and sleep duration was defined as the difference in time between the loss and recovery of the righting reflex.

The caffeine group was given pentobarbital sodium 30 min before the sleep coordination experiment. The tail of the mouse was lifted and the mouse was placed belly up on a hot plate at 37 °C. The time when the righting reflex disappeared was recorded (the time when the righting reflex disappeared to the time when the righting reflex reappeared). The mice whose righting reflex was restored were placed in the back position immediately after the first righting. If the mice turned over automatically within 30 s, the previous time was the recovery time; otherwise, the time of the second righting was taken as the duration of sleep. The sleep latency period (from the injection of pentobarbital sodium to the disappearance of righting reflex) and sleep duration were recorded, respectively. This test was used to measure sleep latency and duration, which are key indicators of daytime sleepiness and hypersomnia.

#### 2.3.2. Open Field Experiment

This experiment adopted specifications for an open field comprising a 50 × 50 cm white square board with 40 cm tall white wall panels on all four sides. Prior to the experiment, the open field’s base was disinfected using 75% alcohol, and after it had dried, the experimental mice were placed at the exact center of the open field. Their activities were then recorded for a duration of 5 min. The activities of the mice in the open field were analyzed using software from Shanghai Xin (Shanghai, China). Following the completion of each animal’s experiment, residual urine and feces within the open field were wiped clean, and the area was sanitized with 75% alcohol to eliminate any olfactory interference from the preceding mouse. This test assessed general locomotor activity and anxiety levels. It was used to evaluate symptoms associated with fatigue and decreased motor capacity during daytime dysfunction.

#### 2.3.3. Morris Water Maze (MWM)

The impact on spatial memory ability in rats or mice was evaluated using the Morris water maze (MWM). All groups of rats or mice underwent a 5 or 6-day spatial navigation experiment, where animals were released from three different quadrants in each training session. The location of the fixed platform was consistent during this period, and the latency from entering the water to finding the safe platform was recorded. The trials were limited to 60 s, with any duration exceeding this limit recorded as the maximum swim time. Training sessions were consistently conducted at a fixed time each day. Following training, tests were administered to gauge the consolidation of memory. With the platform removed, animals were released from any random quadrant and their swimming paths were tracked for 60 s. Metrics such as the time spent and distance traveled in the target quadrant where the platform had been located, the number of times the platform location was crossed, and the initial time taken to find the platform location during training were calculated. This test evaluated spatial learning and memory. It was used to evaluate symptoms associated with cognitive and memory decline during daytime dysfunction.

#### 2.3.4. Rotating Rod Test

The rotarod test was employed to assess motor coordination and balance in the rodents. The first day, all mice underwent a 5 min training session on the rotarod, during which they were promptly repositioned on the rod whenever they fell. The second day, the latency to fall and the distance covered before falling were recorded for all mice. This test evaluated motor coordination and balance. It was used to evaluate symptoms associated with decreased motor coordination during daytime dysfunction.

#### 2.3.5. Grip Test

The primary objective of the grip strength test in mice is to evaluate their muscle function and neurological condition. The mouse was placed on a grid, with its trunk maintained horizontally, allowing only its front paws to grasp the grid before any measurements were taken. Gently pulling back on the mouse’s tail ensured that the mouse held onto the top of the grid with its trunk remaining horizontal. The maximum grip force value displayed on the screen was then recorded. This procedure was repeated for a total of three trials. This test measured muscle strength and endurance, and was used to evaluate symptoms associated with decreased motor coordination during daytime dysfunction.

### 2.4. Physiological Studies

After multiple-factor sleep deprivation, rats were anesthetized, and blood samples were collected from the abdominal aorta. A portion of the blood was collected into heparinized tubes for complete blood count testing, while another portion was collected into plain tubes for biochemical testing. The plain collection tubes without additives were allowed to stand at room temperature for 90 min, followed by centrifugation at 4000 revolutions per minute for 20 min using a low-temperature high-speed centrifuge (Eppendorf 5810R, Hamburg, Germany) to separate the serum, which was then collected for biochemical analysis. Meanwhile, the whole blood containing anticoagulant was processed through an automated hematology analyzer (XN-1000V, Sysmex, Kobe, Japan) for a complete blood count. The serum biochemical parameters were measured using an automatic biochemistry analyzer (Cobas c311, Roche, Mannheim, Germany), employing commercial reagent kits.

### 2.5. ELISA

Hippocampal tissues from mice were harvested and weighed, followed by homogenization in phosphate buffered saline (PBS) at a ratio of 1:10. The homogenate was then centrifuged at 4 °C, 5000× *g* for 15 min to collect the supernatant. Protein concentrations in the supernatants were determined using a BCA Protein Assay Kit according to the manufacturer’s instructions. Measurements of SOD, GSH, MDA, IL-6, and TNF-αlevels were all carried out in accordance with the provided protocols or kit instructions.

Mouse whole blood was allowed to rest at room temperature for one hour to facilitate layer separation. Following this, the sample was centrifuged at (4 °C, 3000× rcf) for 15 min to collect the supernatant. The measurement of S100β levels in the collected supernatant was then conducted according to the provided protocol or kit instructions.

Hypothalamic tissues from rats were obtained and weighed, subsequently homogenized in phosphate buffered saline (PBS) at a ratio of 1:10. The homogenate was centrifuged at 4 °C, 5000× *g* for 15 min to isolate the supernatant. Protein concentrations in each supernatant sample were determined using a BCA Protein Assay Kit following the manufacturer’s guidelines. The measurement of serotonin 5-HT levels was carried out in accordance with the provided instructions.

Hippocampal tissues from rats were harvested and weighed, followed by homogenization in phosphate buffered saline (PBS) at a ratio of 1:10. The homogenate was then centrifuged at 4 °C, 5000× *g* for 15 min to collect the supernatant. Protein concentrations in the supernatants were determined using a BCA Protein Assay Kit according to the manufacturer’s instructions. Measurements of IL1-β, IL-6, TNF-α, and MDA levels were all carried out in accordance with the provided protocols or kit instructions.

Rat whole blood was allowed to rest at room temperature for one hour to facilitate layer separation. Following this, the sample was centrifuged at (4 °C, 3000× rcf) for 15 min to collect the supernatant. The measurement of S100βCRH, ACTH, and CORT levels in the collected supernatant was then conducted according to the provided protocol or kit instructions.

### 2.6. The DSI Fully Implantable Telemetry System Enables the Monitoring of Sleep Patterns in Animals While They Are Awake and Behaving Naturally

The classic sodium pentobarbital-induced sleep test can only assess parameters such as sleep latency and duration, but it does not allow for the monitoring or differentiation of sleep architecture.

The DSI fully implanted telemetry system can record and analyze biological signals such as an electroencephalogram (EEG), electromyogram (EMG), etc., enabling further analysis of sleep structure including paradoxical sleep (PS), slow-wave sleep (SWS), wakefulness, and active wakefulness.

Preoperative preparation: After one week of adaptive feeding, SD rats underwent implantation surgery and were placed in an oxygen chamber containing 4% isoflurane (flow rate 4 L/min) until the disappearance of reflex responses. Subsequently, they were removed and placed on a sterile platform of a stereotaxic apparatus, where anesthesia was maintained via a nose cone with 1.5% isoflurane in oxygen (flow rate 4 L/min) with spontaneous breathing. During the surgery, to prevent hypothermia in the rats, a heating pad was placed beneath the surgical stage. The hair was shaved from the head and neck region, and the surgical area was disinfected using 75% alcohol followed by iodophor.

Implant fixation: A 2.5 to 3.5 cm incision was made along the midline from the rat’s forehead to the neck. The skin on the back was bluntly dissected, and the HD-S02 type small animal radio–telemetric physiological signal implant was placed subcutaneously on the rat’s back. For electroencephalogram (EEG) recordings, two electrode wires were directed cranially. The bregma point was located, and marks were made 2 mm to either side of it using a scalpel. Holes were drilled through the skull without damaging the brain tissue. Stainless steel screw electrodes with wire wrapped around them were secured in these holes, and dental cement was used to fix them in place. For electromyogram (EMG) recordings, two electrodes were inserted bilaterally into the neck sternocleidomastoid muscles using a 22 G needle, and they were secured in place using single-stitch sutures. Following the surgery, with appropriate postoperative care, the rats were allowed to recover for 7 days, and antibiotics were administered continuously for 3 days post-surgery. Sleep monitoring commenced from 12:00 a.m.

Data acquisition and analysis: The NeuroScore 3.1.1 software’s default rodent sleep analysis module was employed to process the electroencephalogram (EEG) data. The analog signals were digitized at intervals of 10 s for further analysis and data export. Typically, based on EEG characteristics, raw signals are classified into distinct sleep stages, comprising wakefulness, active waking (a state of wakefulness with movement), slow-wave sleep (SWS), and paradoxical sleep (PS), also known as paradoxical sleep. This categorization enables a detailed assessment of the sleep architecture throughout the monitoring period. DSI telemetry systems were utilized to continuously monitor and record sleep patterns, providing real-time data on sleep architecture. This helped us to assess the quality and quantity of sleep, which are important components of daytime dysfunction.

### 2.7. RNA Preparation and Sequencing

Rat hypothalamic tissue mRNA profiles were generated by deep sequencing on the Illumina® (#E7530L, Ageilent Technologies, Santa Clara, CA, USA) platform. Differential expression genes were selected via correlation testing, principal component analysis, and cluster analysis, in which a fold change (FC) greater than 1.5 and *p*-value less than 0.05 could be regarded as significant. Differentially expressed genes (DEGs) were clustered into pathways of hypothalamic tissue through KEGG (https://www.genome.jp/kegg/, accessed on 18 May 2024).

### 2.8. Western Blot

From the hypothalamic and hippocampal tissues of rats, total protein was extracted using RIPA lysis buffer supplemented with protease inhibitors (1:100) and phosphatase inhibitors (1:100). The extracted total protein was then centrifuged at 12,000 rpm for 15 min at 4 °C, after which the supernatant was carefully aspirated. The protein concentration in the collected supernatant was determined following the instructions provided with the BCA assay kit. The supernatant was mixed with Protein Sample Loading Buffer at a ratio of 1:4 and denatured at 95–100 °C in a metal bath for 15 min. Samples (25 μg) were then separated by electrophoresis on 7.5–12.5% SDS-PAGE gel and subsequently transferred onto a nitrocellulose membrane via wet blotting. After blocking the protein with 5% non-fat milk, the blot was incubated with the primary antibody at 4 °C for 15 h. The membrane was washed three times with TBST for 15 min each, followed by incubation with a horseradish peroxidase-conjugated secondary antibody at room temperature for 1.5 h. Protein expression levels were visualized using enhanced chemiluminescence.

### 2.9. Statistics

All the data are presented as mean ± SD or mean ± SEM. Statistical analyses were performed on GraphPad Prism version 9 (GraphPad Software, La Jolla, CA, USA). Statistical significance was calculated with a one-way ANOVA corrected for multiple comparisons, represented in all figures as follows: * *p* < 0.05, ** *p* < 0.01, *** *p* < 0.001, **** *p* < 0.0001, # *p* < 0.05, ## *p* < 0.01, ### *p* < 0.001, #### *p* < 0.0001.

## 3. Results

### 3.1. Single-Factor Sleep Deprivation Prolongs Sleep Duration in Mice

**Figure 1:**
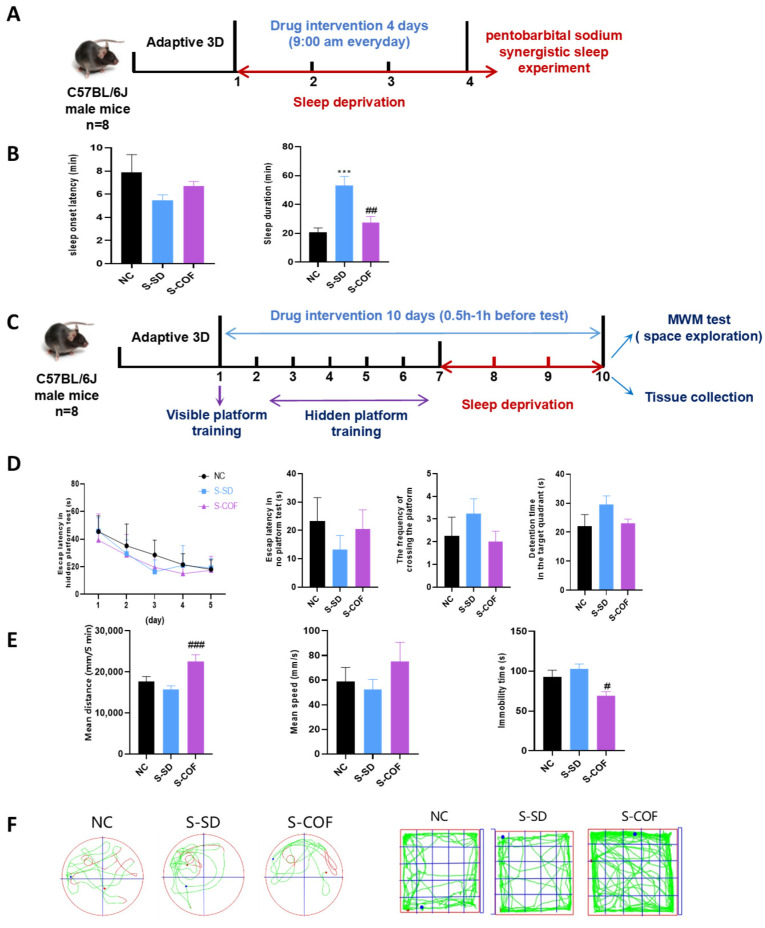
The effects of single-factor sleep deprivation on sleep patterns, memory capabilities, and activity levels in mice: (**A**) Experimental design diagram; (**B**) Pentobarbital sodium synergistic sleep experiment: sleep onset latency, sleep duration; (**C**) Morris water maze schematic used in the experimental design; (**D**) MWM experimental results: escape latency in no platform test, number of platform crossings, detention time in the target quadrant; (**E**) Open field test: mean distance, mean speed, immobility time; (**F**) representative swimming tracks of mice, representative activity tracks of mice. *** *p* < 0.005 vs. NC group. # *p* < 0.05, ## *p* < 0.01, ### *p* < 0.001 vs. S-SD group.

The experimental design involved adaptively feeding mice for three days followed by 72 h of single-factor sleep deprivation (rotating-rod sleep deprivation) (S-SD) using the rotating-rod method. After the sleep deprivation period, intragastric administration was performed, and behavioral experiments were conducted (Figure 1A).

Pentobarbital sodium sleep synergy experiments: The S-SD (sleep-deprived) group exhibited a notable increase in sleep duration, with an average extension of 32.5 min compared with the NC group (*p* < 0.001). The administration of caffeine significantly reduced sleep duration in the S-SD group, resulting in an average decrease of 25.7 min (*p* < 0.01) compared with the S-SD group (Figure 1B).

Morris water maze (MWM): The Morris water maze (MWM) is a test used to assess spatial awareness, orientation, and memory in mice. During the water maze experiment, oral administration of the drug was started 6 days before modeling and continued for 10 days (once a day, 9:00 am). After 6 days of hidden platform training, the single-factor sleep deprivation model was constructed, and the space exploration experiment was conducted within 0.5–1 h after the animals left the sleep deprivation instrument (Figure 1C). In the spatial exploration experiment, the S-SD group did not differ significantly from the control group in discovering the hidden platform, time spent in the target quadrant, or number of platform crossings. Single-factor sleep deprivation (rotating-rod sleep deprivation) did not significantly affect spatial memory abilities (*p* > 0.05) (Figure 1D).

Open field: The open field test was utilized as a means of assessing spontaneous behavior and investigating behavior and stress levels in a novel environment. In comparison to the NC group, the S-SD group did not exhibit statistically significant alterations in total distance traveled, average speed, or immobility duration (*p* > 0.05) (Figure 1E).

In summary, single-factor sleep deprivation resulted in prolonged sleep duration and signs of sleepiness but did not significantly affect motor abilities, emotional state, cognitive function, or activity levels in the tested mice. These findings contribute to our understanding of the effects of acute sleep deprivation on sleep patterns and behavior in mice.

### 3.2. Multi-Factor Sleep Deprivation Prolongs Sleep Duration and Significantly Impairs Learning and Memory Abilities in Mice

**Figure 2:**
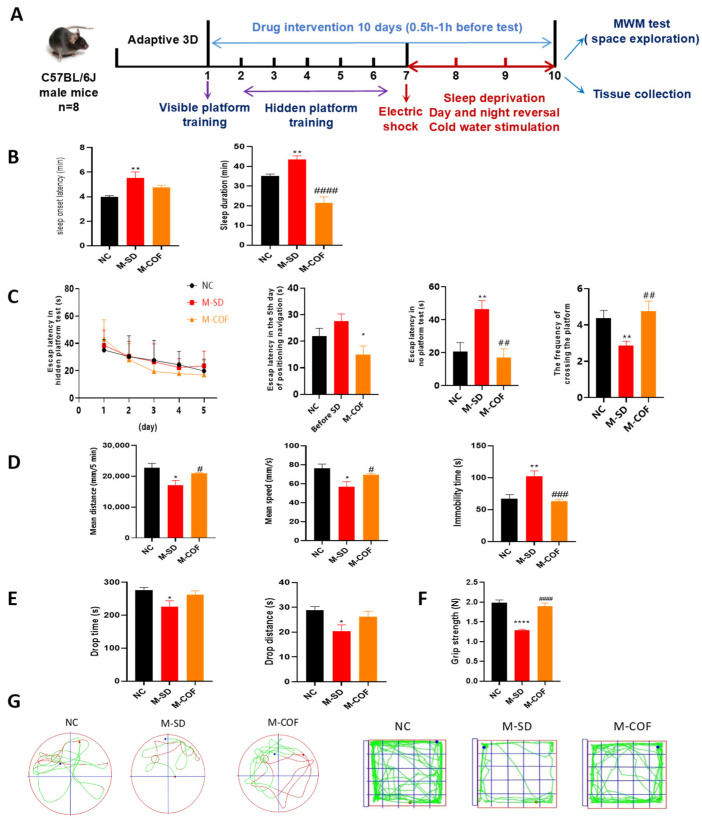
The effects of multi-factor sleep deprivation on sleep patterns, memory capabilities, activity levels, balance, and grip in mice: (**A**) Experimental design diagram; (**B**) Pentobarbital sodium synergistic sleep experiment: sleep onset latency, sleep duration; (**C**) MWM experimental results: time finding the platform, escape latency in no-platform test, number of platform crossings, detention time in the target quadrant; (**D**) Open field test: mean distance, mean speed, immobility time; (**E**) Baton twirling test: drop time, drop distance; (**F**). Grip test: grip strength; (**G**) representative swimming tracks of mice, representative activity tracks of mice. * *p* < 0.05, ** *p* < 0.01, **** *p* < 0.001 vs. NC group. # *p* < 0.05, ## *p* < 0.01, ### *p* < 0.001, #### *p* < 0.001 vs. M-SD group.

In the experimental procedure illustrated in Figure 2A, oral administration of the drug was started 6 days before modeling and continued for 10 days (once a day, 9:00 am). After 5 days of hidden platform training, the multi-factor sleep deprivation model was constructed, and the space exploration experiment was conducted within 0.5–1 h after the animals left the sleep deprivation instrument (Figure 2A).

The experimental procedure illustrated in Figure 2A involved a three-day adaptive feeding regimen for mice, followed by a 72 h period of multi-factor sleep deprivation, which included a reversed light–dark cycle, daily cold water stimulation, and electrode stimulation at 0.5 mA/2 s intervals. Following the sleep deprivation phase, intragastric administration was carried out, and behavioral experiments were conducted 0.5–1 h post-administration (Figure 2A).

Pentobarbital sodium sleep synergy experiments: Compared with the NC group, the sleep time of the M-SD group was significantly extended by 15.18 min (*p* < 0.01). Compared with the M-SD group, the sleep time of the caffeine group was significantly shortened by 23.26 min (*p* < 0.001) (Figure 2B).

Morris water maze (MWM): The Morris water maze (MWM) experiment assesses spatial learning and memory in mice. Over 6 days, mice improved in finding a hidden platform, showing short-term memory formation. On the 5th day, mice given caffeine had significantly decreased escape latency, suggesting improved learning abilities (*p* < 0.05) (Figure 2C). In the spatial exploration experiment, compared with the NC group, the M-SD (multi-factor sleep-deprived) group showed an increased time to first discover the hidden platform (*p* < 0.01) and a significant decrease in platform crossings (*p* < 0.05). This indicates that multi-factor sleep deprivation significantly impaired spatial memory capabilities. Following treatment with caffeine (COF), the M-SD group exhibited a significant decrease in time to first discover the hidden platform (*p* < 0.01) and an increase in platform crossings (*p* < 0.01) (Figure 2C).

Open field: Compared with the NC group, the multi-factor sleep-deprived group exhibited significantly reduced total distance moved (*p* < 0.05), decreased average speed (*p* < 0.05), and increased immobility time (*p* < 0.05). This indicates that multi-factor sleep deprivation reduced their motor capabilities and induced depression-like behavior. After caffeine treatment, the total movement distance increased (*p* < 0.05), the total average speed increased (*p* < 0.05), and the time spent immobile decreased significantly (*p* < 0.001) (Figure 2D).

Rotary rod test: Compared with the NC group, the falling distance and falling time of the M-SD group were decreased (*p* < 0.05), indicating that the motor coordination ability of M-SD group was decreased due to multi-factor sleep deprivation. The falling distance and falling time after COF increased to a certain extent, but there was no significant difference (Figure 2E).

Grip test: After the modeling, the gripping power of the M-SD group was significantly decreased compared with that of the NC group (*p* < 0.05). After caffeine treatment, the gripping power of the mice was significantly improved (*p* < 0.05) (Figure 2F).

The multi-factor sleep deprivation (rotating-rod sleep deprivation) model showed significantly prolonged duration, reduced physical strength, and damaged cognitive functions. This model can effectively mimic clinical symptoms of daytime functional impairments like excessive sleepiness, cognitive deficits, and altered mood states.

### 3.3. Multi-Factor Sleep Deprivation Affects Oxidative Stress, Inflammatory Factors, and the Blood–Brain Barrier in Mice

Sleep disorders are a major cause of daytime dysfunction, which inflicts multi-faceted damage to the body. Cognitive dysfunction is the main manifestation of daytime dysfunction, and its cognitive decline is closely related to oxidative stress in hippocampal tissue, inflammatory response, and blood–brain barrier permeability.

**Figure 3:**
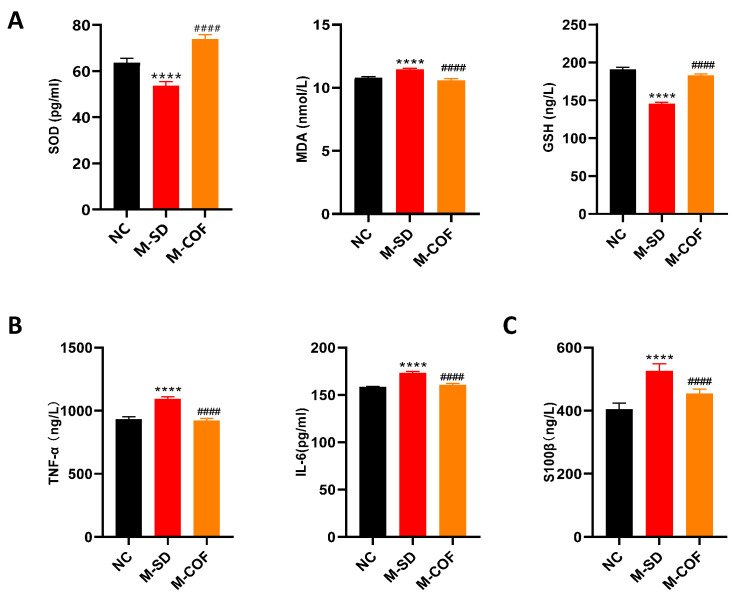
The impacts of multi-factor sleep deprivation on oxidative stress, inflammatory cytokines, and blood–brain barrier biomarkers in mice: (**A**) Oxidative stress levels: SOD, MDA, GSH; (**B**) Inflammatory factor levels: TNF-α, IL-6; (**C**) Blood–brain barrier marker: S100β. **** *p* < 0.0001 vs. NC group. #### *p* < 0.0001 vs. M-SD group.

Compared with the NC group, mice subjected to multi-factor sleep deprivation (rotating-rod sleep deprivation) exhibited significantly reduced levels of SOD and GSH (*p* < 0.0001), as well as notably increased levels of MDA (*p* < 0.0001). Following treatment with caffeine, there was a significant increase in the levels of SOD and GSH (*p* < 0.0001), and a pronounced decrease in MDA levels (*p* < 0.0001) (Figure 3A).

Compared with the NC group, the contents of TNF-α and IL-6 in the M-SD mice were significantly increased (*p* < 0.0001) (Figure 3B).

Compared with the NC group, the M-SD group exhibited a statistically significant elevation in serum S100β levels (*p* < 0.0001) (Figure 3C).

Following treatment with caffeine, there was a significant increase in the levels of SOD and GSH (*p* < 0.0001), and a pronounced decrease in MDA levels (*p* < 0.0001) (Figure 3A). Additionally, the contents of TNF-α and IL-6 were significantly reduced (*p* < 0.0001) (Figure 3B). Furthermore, a significant decrease in serum S100β levels was observed (*p* < 0.0001) (Figure 3C).

In summary, multi-factor sleep deprivation resulted in oxidative stress, inflammation, and blood–brain barrier disruption, which are key factors contributing to cognitive decline. Caffeine treatment significantly mitigated these effects, highlighting its potential as a therapeutic agent for sleep disorder-related cognitive impairments.

### 3.4. Multi-Factor Sleep Deprivation Changes Sleep Duration and Sleep Structure in Rats

The classical sodium pentobarbital sleep experiment can measure the duration of sleep, but its inability to detect the structure of the subject’s sleep, and thus the quality of their sleep, cannot be evaluated. Therefore, we used the DSI fully implantable telemetry system to detect the sleep of animals in the awake and natural state.

**Figure 4:**
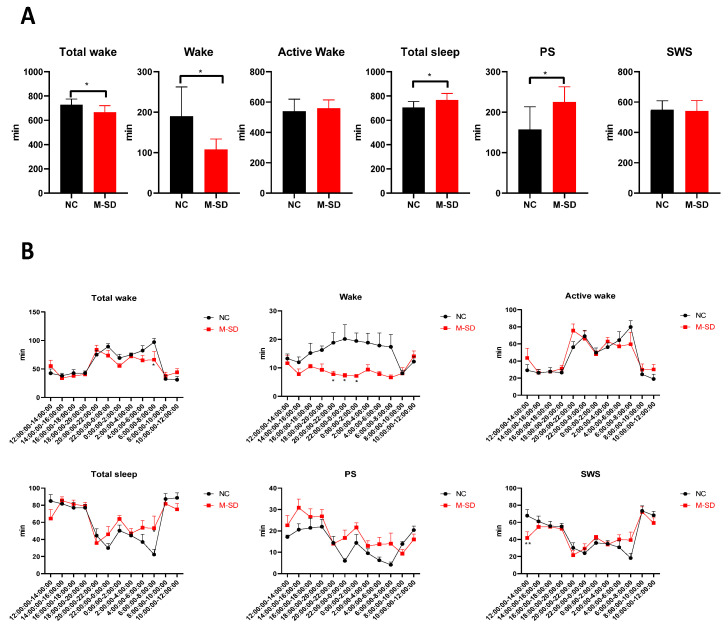
Sleep duration and sleep structure of multi-factor sleep-deprived rats: (**A**) Sleep duration: total waking, waking, active waking, total sleep, PS, SWS; (**B**) Sleep structure: total waking, waking, active waking, total sleep, PS, SWS. * *p* < 0.05, ** *p* < 0.01 vs. NC group.

Sleep duration and structure in rats were significantly altered following 72 h of multi-factor sleep deprivation. Compared with the NC group, the sleep time and sleep structure of rats were significantly changed; the total waking time and waking time were reduced (*p* < 0.05), total sleep time increased (*p* < 0.05), and paradoxical sleep time increased (*p* < 0.05) (Figure 4A). According to the analysis of sleep structure, the overall waking time of the model animals decreased significantly in the period from 6:00 to 8:00 (before the light was turned on) (*p* < 0.05), and the waking time decreased significantly in the period from 20:00 to 2:00 (after the light was turned off) (*p* < 0.05). The overall sleep time was significantly increased between 6:00 and 8:00 (before the light was turned on) (*p* < 0.05), while slow-wave sleep was significantly decreased between 12:00 and 14:00 (*p* < 0.05) (Figure 4B). The results show that multi-factor sleep deprivation can greatly impact sleep structure, duration, and quality, leading to symptoms like daytime sleepiness and fatigue similar to daytime dysfunction.

### 3.5. Multi-Factor Sleep Deprivation Affects Physiological and Biochemical Indexes in Rats

Relevant physiological and biochemical indexes of animal models were tested to evaluate the effects of multi-factor sleep deprivation.

Blood routine indexes: there were no significant differences in blood routine indexes such as red blood cells and white blood cells between the multi-factor sleep deprivation (M-SD) group and the NC group (Table 1).

Liver function: compared with the NC group, the M-SD group showed significantly increased levels of ALT, AST, and TP (*p* < 0.05) (Table 2), indicating abnormal liver function.

Renal function: compared with the NC group, the level of CRE in the M-SD group was increased (*p* < 0.05) (Table 2), suggesting abnormal effects on renal function.

Metabolic function: compared with the NC group, the level of LACT in the M-SD group was significantly increased (*p* < 0.05) (Table 2), suggesting metabolic dysfunction.

In summary, multi-factor sleep deprivation resulted in significant alterations in liver and renal function, as well as metabolic dysfunction, highlighting the importance of adequate sleep for maintaining physiological homeostasis.

### 3.6. Multi-Factor Sleep Deprivation Impairs Learning and Memory Ability in Rats

**Figure 5:**
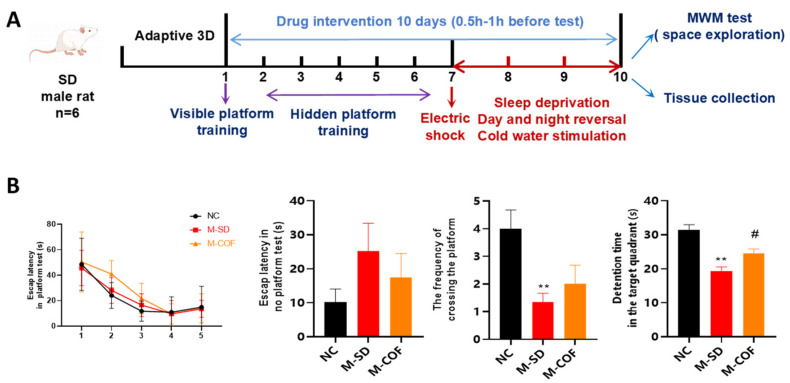
The effects of multi-factor sleep deprivation on memory capabilities in rats: (**A**) Experimental design diagram; (**B**). MWM experimental results: time finding the platform, escape latency in no-platform test, number of platform crossings, detention time in the target quadrant. ** *p* < 0.01, vs. NC group. # *p* < 0.05, vs. M-SD group.

The experimental workflow shows that SD rats were fed for three days and trained in MWM for five days. Over time, all groups of rats took less time to find the hidden platform, showing the development of short-term memory (Figure 5A).

In the space exploration experiment, compared with the NC group, the multi-factor sleep deprivation group had a significant decrease in the number of times crossing the platform and the time staying in the target quadrant (*p* < 0.01), indicating that multi-factor sleep deprivation significantly reduced their spatial memory ability. After the intervention of caffeine, the residence time in the target quadrant was significantly increased (*p* < 0.05). These findings suggest that caffeine protected and ameliorated impairments in spatial memory and learning exploration ability in multi-factorially sleep-deprived animals (Figure 5B).

### 3.7. Multi-Factor Sleep Deprivation Affects Oxidative Stress, Inflammatory Factors, Blood–Brain Barrier, and Hypothalamic Axis in Rats

**Figure 6:**
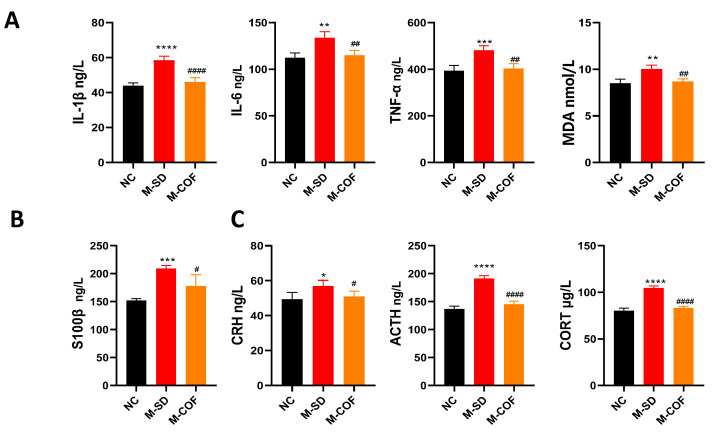
The impacts of multi-factor sleep deprivation (rotating-rod sleep deprivation) on inflammatory cytokines, oxidative stress, blood–brain barrier biomarkers, and HPA axis in rats: (**A**) Inflammatory factor levels and oxidative stress: IL-1β, IL-6, TNF-α, MDA; (**B**) blood–brain barrier marker: S100β; (**C**) PA axis levels: CRH, ACTH, CORT. * *p* < 0.05, ** *p* < 0.01, *** *p* < 0.001, **** *p* < 0.0001 vs. NC group. # *p* < 0.05, ## *p* < 0.01, #### *p* < 0.0001 vs. M-SD group.

Inflammatory factors: compared with the NC group, rats subjected to multi-factor sleep deprivation had markedly increased levels of TNF-α and IL-6 (*p* < 0.0001) (Figure 6A).

Oxidative stress: compared with the NC group, rats subjected to multi-factor sleep deprivation exhibited significantly increased levels of MDA (*p* < 0.0001) (Figure 6A).

Blood–brain barrier: Compared with the NC group, the serum S100β levels in rats subjected to multi-factor sleep deprivation were significantly elevated (*p* < 0.0001) (Figure 6B).

HPA axis: compared with the NC group, multi-factor sleep deprivation led to hyperfunction of the HPA axis, with significantly increased levels of CRH, ACTH, and CORT (*p* < 0.0001) (Figure 6C).

Multi-factor sleep deprivation also resulted in hyperfunction of the HPA axis, as evidenced by significantly increased levels of CRH, ACTH, and CORT. Treatment with caffeine significantly alleviated the hyperfunction of the HPA axis, indicating a potential role in reducing stress responses induced by sleep deprivation.

### 3.8. Effects of Multi-Factor Sleep Deprivation on Transcriptome and Bioinformatics in the Hypothalamic Region in Rats

**Figure 7:**
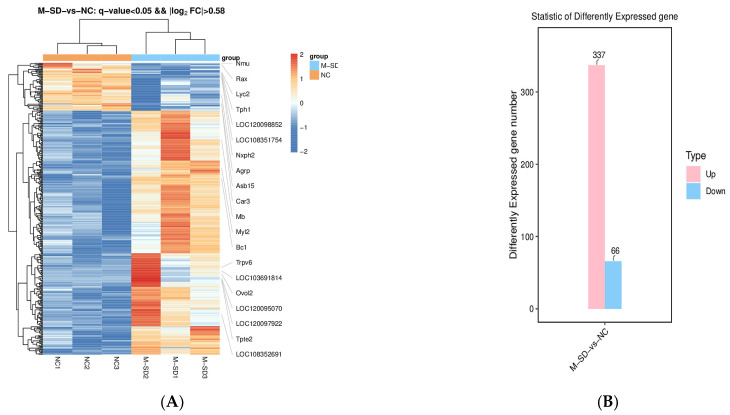

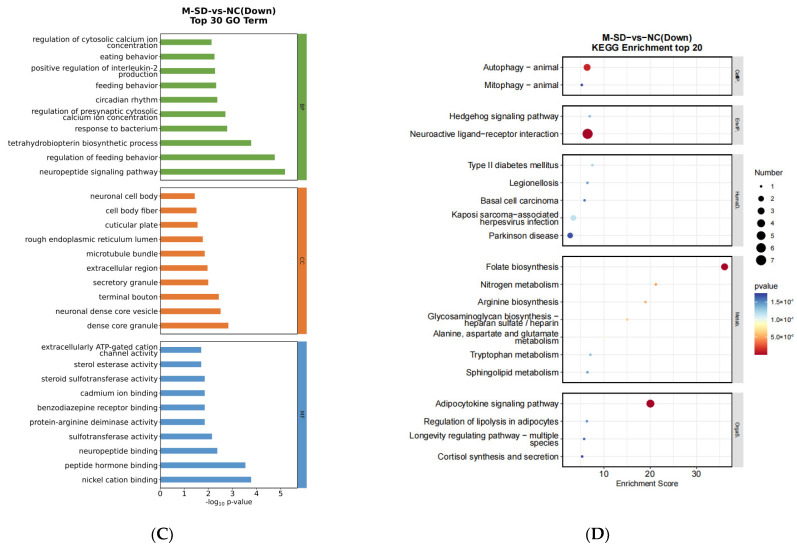
Transcriptome sequencing analysis of rat hypothalamic tissue after multi-factor sleep deprivation: (**A**) Hierarchical clustering of all the DEGs was based on the log10RPKM values; (**B**) Quantification of significantly up- and downregulated genes in NC vs. M-SD; (**C**) Quantification of significantly up- and downregulated genes in NC vs. M-SD; (**D**) GO enrichment analysis; (**D**): KEGG enrichment analysis.

To investigate the effects of multi-factor sleep deprivation on gene expression changes in the rat hypothalamus, high-throughput sequencing was performed on the Illumina platform. Cluster analysis of the samples based on DEGs showed that the multi-factor sleep groups had better gene separation than the control group (Figure 7A).

Data from the two groups were analyzed for differences using DESeq2 software version 1.12.3 (with Log2FC ≥ 1) as a screening criterion. The statistical results showed that there were 337 differential genes in the multi-factor sleep deprivation group compared with the blank control group, of which 337 genes were upregulated and 66 genes were downregulated in expression (Figure 7B).

In order to explore the changes in biological function of DEGs caused by multi-factor sleep deprivation, we conducted GO enrichment analysis and KEGG enrichment analysis on DEGs and found associations with circadian rhythm, neuropeptide signaling pathways, and neuroactive ligand–receptor interaction (Figure 7C,D).

According to the results of the transcriptome, multi-factor sleep deprivation leads to significant alterations in biological functions related to circadian rhythms, as well as in signaling pathways such as neuropeptide signaling and neuroactive ligand–receptor interaction. Based on these findings, our subsequent investigations into the multi-factor sleep deprivation animal model will focus on the following areas: circadian rhythms and neuroactive ligand–receptor interaction.

### 3.9. Multi-Factor Sleep Deprivation Affects Circadian Rhythm

**Figure 8:**
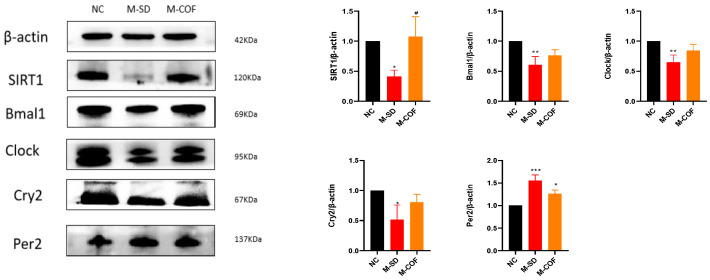
The impacts of multi-factor sleep deprivation on proteins associated with the circadian rhythm in rats: SIRT1, Bmal1, Clock, Cry2, Per2 protein expression levels in hypothalamus tissue. * *p* < 0.05, ** *p* < 0.01, *** *p* < 0.005 vs. NC group. # *p* < 0.05, vs. M-SD group.

Expression levels of circadian rhythm proteins: compared with the NC group, rats subjected to multi-factor sleep deprivation (M-SD) had significantly reduced expression levels of Sirt1, Bmal1, Clock, and Cry2 (*p* < 0.05), and significantly increased expression levels of Per2 protein (Figure 8).

Pharmacological intervention with caffeine: following pharmacological intervention with caffeine, the hypothalamic expression levels of Bmal1, Clock, and Cry2 proteins all significantly increased, while the expression level of Per2 decreased (Figure 8).

The results indicate that multi-factor sleep deprivation (M-SD) led to a disruption of the circadian rhythm, as evidenced by altered expression levels of key circadian rhythm proteins. Specifically, the expression levels of Sirt1, Bmal1, Clock, and Cry2 were significantly reduced, while the expression level of Per2 was significantly increased.

In summary, multi-factor sleep deprivation results in significant alterations in the expression of circadian rhythm proteins, leading to disrupted sleep quality and daytime dysfunction.

### 3.10. Multi-Factor Sleep Deprivation Affects Sleep–Wake-Related Neurotransmitters and Their Receptors in Rats

**Figure 9:**
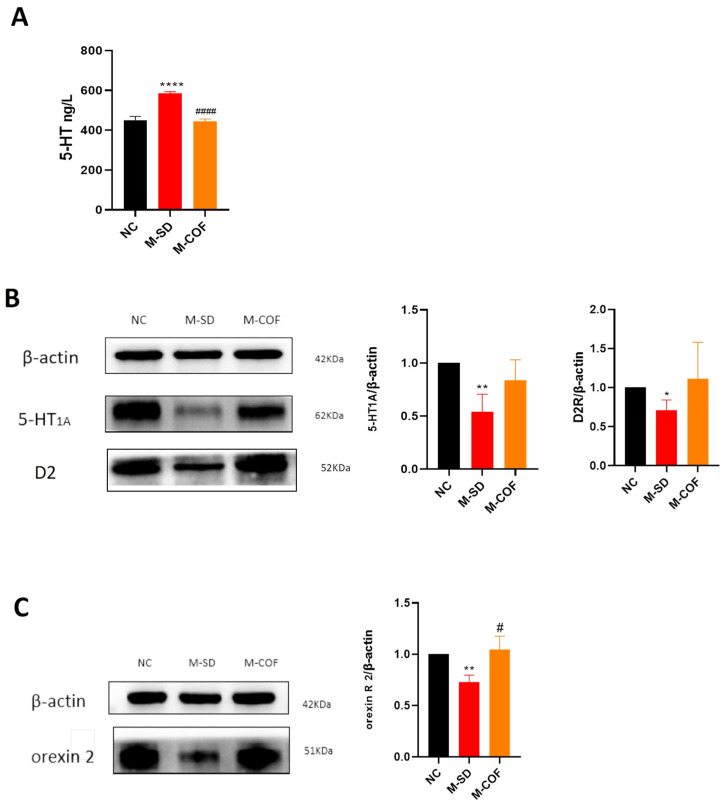
Effects of multi-factor sleep deprivation on sleep–wake-related neurotransmitters and their receptors in rats: (**A**) levels of 5-ht in hypothalamic tissues; (**B**). 5-HT1A, D2A protein expression levels in hypothalamus tissue; (**C**) orexin 2 R protein expression levels in hypothalamus tissue. * *p* < 0.05, ** *p* < 0.01, **** *p* < 0.0001 vs. NC group. # *p* < 0.05, #### *p* < 0.0001 vs. M-SD group.

5-HT neurotransmitter content: compared with the NC group, rats subjected to multi-factor sleep deprivation (M-SD) had a significant increase in 5-HT content (*p* < 0.05) (Figure 9).

5-HT1A receptor protein expression: the protein expression of the 5-HT1A receptor was notably decreased in the M-SD group (*p* < 0.05) (Figure 9).

D2A receptor protein expression: the protein expression of the D2A receptor was notably decreased in the M-SD group (*p* < 0.05) (Figure 9).

Orexin receptor 2 protein expression: multi-factor sleep deprivation resulted in a decreased protein expression of orexin receptor 2 (*p* < 0.05) (Figure 9).

Pharmacological intervention with caffeine: after intervention with caffeine, the 5-HT content significantly decreased (*p* < 0.05) (Figure 9), and expression of orexin receptor 2 significantly increased (*p* < 0.05) (Figure 9).

Multi-factor sleep deprivation resulted in significant alterations in the levels of the 5-HT neurotransmitter and its receptors, as well as dopamine D2 and orexin receptor 2 expression, leading to disruptions in sleep–wake regulation and contributing to hypersomnia symptoms. Caffeine treatment significantly mitigated these effects, highlighting its potential as a therapeutic agent for sleep disorder-related disruptions in sleep–wake regulation.

### 3.11. Multiple Factors of Sleep Deprivation Affect Adenosine A2A Receptors in Rats

**Figure 10:**
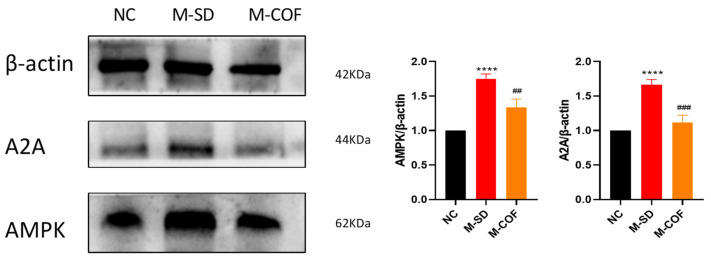
Effects of multi-factor sleep deprivation (bar rotational sleep deprivation) on adenosine receptor 2A-related expression proteins in rat hypothalamus tissue. A2A, AMPK protein expression levels in hypothalamus tissue. **** *p* < 0.0001 vs. NC group. ## *p* < 0.01, ### *p* < 0.005 vs. M-SD group.

Adenosine A2A receptor protein expression: Compared with the NC group, the protein expression of the adenosine A2A receptor significant increased in the M-SD group (*p* < 0.005) (Figure 10). This translation conveys that the protein expression levels of AMPK, a downstream signaling molecule involved in the pathway, were notably increased (*p* < 0.005) (Figure 10).

Pharmacological intervention with caffeine: after intervention with caffeine, the expression of adenosine A2A receptor protein and AMPK protein expression was significantly reduced (*p* < 0.01) (Figure 10).

In summary, multi-factor sleep deprivation results in an increase in adenosine levels in the brain, which can lead to upregulation of adenosine A2A receptors and the downstream signaling pathway for AMPK expression, increasing the need for sleep and feelings of fatigue.

### 3.12. Multiple Factors of Sleep Deprivation Affect Synaptic Plasticity in Rats

**Figure 11:**
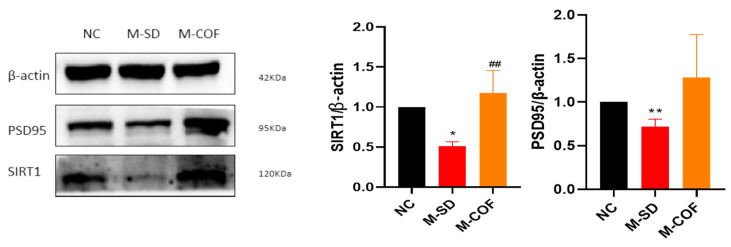
Effects of multi-factor sleep deprivation on memory-related proteins expression in rat hippocampal tissue. SIRT1, PSD95 protein expression levels in hippocampal tissue. * *p* < 0.05, ** *p* < 0.01, vs. NC group. ## *p* < 0.01 vs. M-SD group.

SIRT1 expression: cCompared with the NC group, rats subjected to multi-factorial sleep deprivation (M-SD) had a significant decrease in SIRT1 expression levels in the hippocampal tissue (*p* < 0.05) (Figure 11).

PSD95 expression: the expression of postsynaptic density protein-95 (PSD95) was also significantly reduced in the hippocampus following M-SD (*p* < 0.05) (Figure 11).

Pharmacological intervention with caffeine: after intervention with caffeine, the expression of SIRT1 and PSD95 proteins significantly increased (*p* < 0.05) (Figure 11).

Multi-factor sleep deprivation led to significantly reduced expression of proteins associated with memory cognitive function, such as SIRT1 and PSD95, leading to a decline in cognitive and memory function.

## 4. Discussion

Based on the clinical characteristics of daytime dysfunction, the effectiveness of the model was characterized by the expression of daytime activity levels, emotional state, motor abilities, and cognitive functions. The animal model of daytime dysfunction exhibited reduced activity levels (as indicated by electroencephalogram and electromyogram recordings), depressive mood (assessed in the open field test), decreased motor abilities (measured in the rotarod and grip strength tests), and impaired spatial learning and memory (evaluated in the Morris water maze). The model confirmed that 3 days of multi-factor sleep deprivation was sufficient to achieve the preparation of an animal model of daytime dysfunction, determined through the exploration of conditions for single-factor and multi-factor sleep deprivation. Studies show that single-factor sleep deprivation can induce prolonged sleep time but does not significantly affect memory, cognitive functions, motor abilities, or mood states. Factors such as cold exposure, circadian rhythm disruption, and electrical stimulation are mature inducers of disrupted sleep structure, cognitive function, activity ability, and mood states. By reasonably controlling the modeling conditions (such as electrical stimulation parameters and temperature), the relevant characteristics of the animal model of daytime dysfunction can be realized [18,19,20]. Unlike single-factor sleep deprivation (rotating-rod sleep deprivation), which only increases sleep duration, sleep deprivation compounded with circadian reversal for 72 h, cold water stimulation once daily, and electrode stimulation at 0.5 mA/2 s simulated a daytime dysfunction animal model [21].

Multi-factor sleep deprivation significantly alters both the duration and structure of sleep, suggesting a compensatory mechanism in response to sleep loss, sleepiness that mimics daytime dysfunction. Sleep problems caused by multi-factor sleep deprivation may be related to disturbances in the expression of neurotransmitters and their receptors, the HPA axis, and circadian rhythm-related proteins. The Morris water maze results demonstrated that multi-factor sleep deprivation impaired spatial memory and learning abilities, as evidenced by the increased time to find the hidden platform and a decrease in platform crossings, representing the decline of memory and cognitive function in simulated daytime dysfunction. The decline in memory and cognitive function caused by multi-factor sleep deprivation may be related to oxidative stress, elevated levels of inflammatory factors, and decreased expression of memory-related proteins BDNF, sirt1, and PSD95 in the hippocampus. The open field test results indicated that multi-factor sleep deprivation reduced motor capabilities and induced depression-like behavior. The rotary rod test showed that multi-factor sleep deprivation decreased motor coordination. The grip test results indicated that multi-factor sleep deprivation caused a decline in muscle strength. Thus, clinical manifestations of daytime dysfunction, such as emotional abnormalities and exercise fatigue, were simulated.

Further investigation reveals that these symptoms are closely linked to hippocampal oxidative stress, inflammatory cytokines, changes in blood–brain barrier markers like S100β, and alterations in HPA axis indicators such as CRH, ACTH, and CORT [22]. Our study revealed that Sirt1 significantly influenced clock gene expression in thalamic tissues. Reduced Sirt1 activity or gene deletion disrupts the circadian cycle [6,23,24]. The multi-factor sleep deprivation model reduced the expression of the circadian rhythm protein Sirt1, impacting related proteins and sleep quality, thus affecting daytime function. Sirt1 is crucial for wakefulness and sleep, and its deficiency, whether genetic or acquired, causes wakefulness issues. This deprivation also lowers the expression of receptors in wakefulness neurons [25,26].

Daytime functional impairment is often linked to sleep deprivation and stress, particularly in jobs like those of military personnel, pilots, shift-working doctors and nurses, and long-haul drivers. Individuals in these roles face challenges such as trans-timezone schedules and inverted day–night cycles, leading to increased fatigue, excessive daytime sleepiness, insomnia, and cognitive decline [27,28].

Daytime functional impairment in modern populations is mainly caused by circadian rhythm disruptions and stress [27]. Previous research typically focused on individual symptoms, neglecting secondary symptoms and the holistic view of the organism. To address this, we developed an animal model that simulates the range of symptoms associated with daytime functional impairment. In traditional Chinese medicine, these symptoms are known as “divine fatigue (shenpi)” and “physical exertion (tilao) [14]”. A comprehensive analysis aligns with clinical practice and the holistic view of traditional Chinese medicine, enhancing our understanding of the underlying pathophysiological mechanisms [29]. Our animal model studies reveal a strong link to inflammatory factors and oxidative stress. Our research showed significant changes in inflammatory factors and oxidative stress indicators in the model [30]. We also found increased blood–brain barrier permeability and heightened activity in the hypothalamic–pituitary–adrenal (HPA) axis, with related hormone secretion [22].

**Figure 12:**
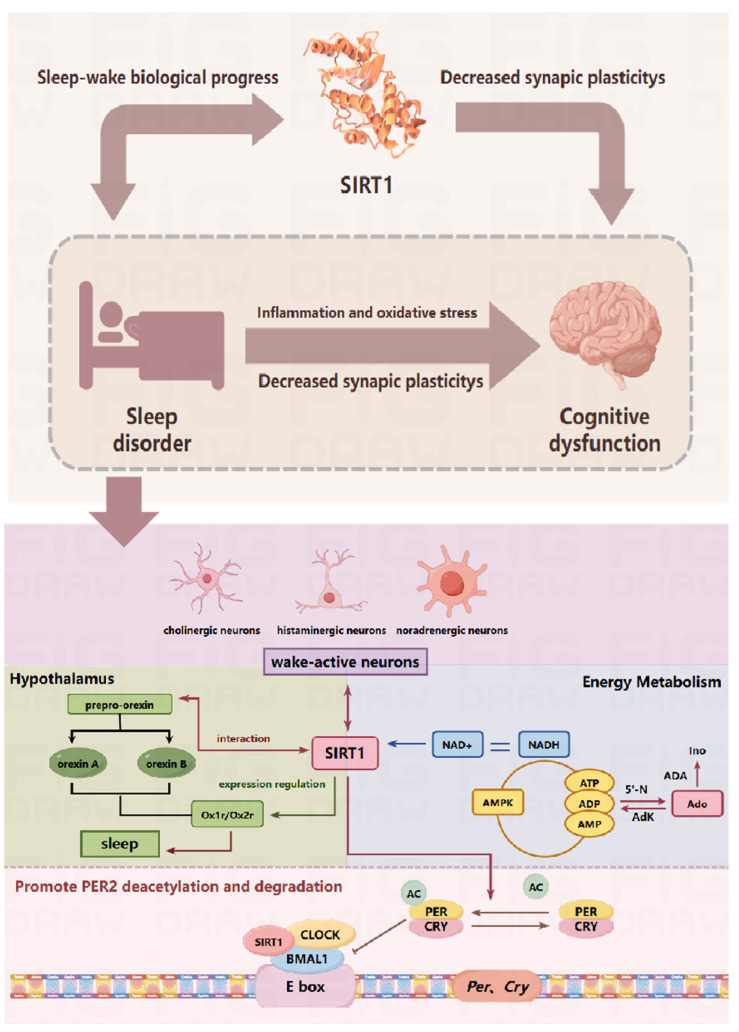
Potential interactions of SIRT1 with sleep–wake progress and memory cognition; intrinsic biological mechanisms of sirt1 regulation of sleep–wakefulness.

Sirt1, a key target in the NAD+-dependent protein deacetylase family, is involved in aging, metabolism, cancer, stress responses, and the biological clock [23,24,31]. ① Circadian rhythm regulation: SIRT1 significantly influences the expression of circadian clock genes, and reduced SIRT1 activity or its genetic removal disrupts circadian rhythms and affects adaptation to new day–night cycles. In a multi-factor sleep deprivation animal model, SIRT1 expression was affected, which further altered the acetylation level of PER, leading to the disturbance of circadian rhythm-related proteins, worsening sleep quality and daytime function [6,32,33,34]. ② Sleep and wake dysfunction: Genetic or acquired reductions in SIRT1 cause severe waking deficits. Sleep deprivation reduces SIRT1 protein levels and wake-promoting neuronal receptors, such as D2R, orexin 2 receptors, and 5-HT1A receptors. [34,35]. ③ Adenosine function: Adenosine and its receptors are crucial for both circadian rhythm and homeostatic sleep drive. Caffeine mediates the sleep–wake pattern through A2A receptors. The adenosine-derived signaling molecule cAMP can influence multiple clock proteins, including SIRT1, via AMP kinase, thereby upregulating this enzyme and affecting downstream pathways. SIRT1 function is also modulated by NAD+ cofactor levels, which are influenced by both circadian and metabolic regulation. [23,25]. ④ Neuroprotective role: SIRT1 plays a significant neuroprotective role in various neurodegenerative diseases, including Alzheimer’s disease (AD). Research shows that SIRT1 levels in the hippocampus of AD patients are notably reduced. Sleep disorders impair cognitive function through neurobiological mechanisms and are common in various diseases. SIRT1 and hippocampal neuronal plasticity are essential for learning and memory [36]. In animal models, multi-factor sleep deprivation reduces SIRT1 and PSD95 protein levels in the hippocampus, suggesting that cognitive dysfunction due to sleep deprivation is linked to decreased SIRT1 protein expression and changes in synaptic plasticity [37] (Figure 12).

Sleep restores energy, protects cognitive function, regulates immune function, and maintains the body’s internal homeostasis. Previous sleep studies often used sodium pentobarbital-induced tests to measure sleep latency and duration. In our study, we used these tests and also the DSI fully implantable telemetry system to evaluate sleep duration and architecture in the animal model. The DSI system allows monitoring of natural sleep patterns in awake, naturally behaving animals, enabling detailed recording and analysis of sleep. Evaluating animal models depends on technological advancements. Future research will explore models under specific conditions using advanced technology.

## 5. Conclusions

Both single-factor and multi-factor sleep deprivation resulted in longer sleep durations in mice. Single-factor sleep deprivation did not affect cognitive function or movement capability, but multi-factor sleep deprivation severely impaired cognitive function and movement capability. Daytime issues from multi-factor sleep deprivation, such as sleepiness, may be linked to reduced protein expression of key SIRT1, along with decreased levels of downstream hypothalamus circadian rhythm-related proteins Bmal1, Clock, and Cry2, and elevated Per2 protein levels, disrupting the expression of proteins involved in the circadian rhythm system loop. Additionally, this is associated with reduced receptor expression of the wakefulness-related neurotransmitters 5-HT1A and orexin 2. The cognitive problems caused by multi-factor sleep deprivation are mainly related to significantly decreased expression levels of memory-related proteins Sirt1 and PSD95 in the hippocampal tissue. The manifestations of multi-factor sleep deprivation align closely with the clinical symptoms of daytime functional disorders, rendering it a stable model for such disorders, useful for drug screening and providing a foundation for studying the biological mechanisms underlying modern health risks.

## Figures and Tables

**Table 1:** Blood routine results of multifactor sleep deprivation.

	WBC (1 × 10^9^/L)	RBC (1 × 10^12^/L)	HGB (g/dL)	PLT (1 × 10^9^/L)	LYMPH (1 × 10^9^/L)	MONO (1 × 10^9^/L)	NEUT (1 × 10^9^/L)	RET (1 × 10^6^/uL)
NC	6.73 ± 2.08	7.78 ± 0.76	15.50 ± 1.36	773.83 ± 221.82	5.91 ± 2.26	0.52 ± 0.20	1.06 ± 0.37	0.41 ± 0.11
M-SD	6.89 ± 1.97	8.72 ± 1.33	16.90 ± 2.69	905.33 ± 335.69	6.24 ± 4.00	0.95 ± 0.51	3.45 ± 3.63	0.36 ± 0.08
M-COF	9.75 ± 3.07	8.83 ± 0.76	17.27 ± 1.41	743.00 ± 161.70	5.90 ± 1.96	0.91 ± 0.32	2.74 ± 1.96	0.38 ± 0.13

**Table 2:** Blood biochemical correlation results of multifactor sleep deprivation.

	ALTL (U/L)	ASTL (U/L)	CREJ2 (umol/L)	CKMB2 (U/L)	LDHI2 (U/L)	TP2 (g/L)	LACT2 (mmol/L)	TRIGL (mmol/L)
NC	44.6 ± 6.3	122.8 ± 18.0	13 ± 2	1331.6 ± 505.7	1069 ± 234	52.8 ± 4.5	4.73 ± 0.47	0.90 ± 0.23
M-SD	78.3 ± 17.6 **	171.6 ± 36.3 *	18 ± 3 **	1511.6 ± 438.7	1355 ± 145	57.9 ± 3.2 *	7.16 ± 1.09 ***	0.73 ± 0.30
M-COF	83.5 ± 12.6	195.9 ± 44.2	18 ± 4	1844.2 ± 538.3	1758 ± 579	61.7 ± 4.2	7.31 ± 1.37	0.82 ± 0.27

* *p* < 0.05, ** *p* < 0.01, *** *p* < 0.001 vs. NC group.

## Data Availability

The data used to support the findings of this study are available from the investigator upon request.
